# The Role of RING Box Protein 1 in Mouse Oocyte Meiotic Maturation

**DOI:** 10.1371/journal.pone.0068964

**Published:** 2013-07-11

**Authors:** Lin Zhou, Ye Yang, Juanjuan Zhang, Xuejiang Guo, Ye Bi, Xin Li, Ping Zhang, Junqiang Zhang, Min Lin, Zuomin Zhou, Rong Shen, Xirong Guo, Ran Huo, Xiufeng Ling, Jiahao Sha

**Affiliations:** 1 State Key Laboratory of Reproductive Medicine, Department of Histology and Embryology, Nanjing Medical University, Nanjing, China; 2 State Key Laboratory of Reproductive Medicine, Nanjing Maternity and Child Health Hospital, Nanjing Medical University, Nanjing, China; Institute of Zoology, Chinese Academy of Sciences, China

## Abstract

RING box protein-1 (RBX1) is an essential component of Skp1-cullin-F-box protein (SCF) E3 ubiquitin ligase and participates in diverse cellular processes by targeting various substrates for degradation. However, the physiological function of RBX1 in mouse oocyte maturation remains unknown. Here, we examined the expression, localization and function of RBX1 during mouse oocyte meiotic maturation. Immunofluorescence analysis showed that RBX1 displayed dynamic distribution during the maturation process: it localized around and migrated along with the spindle and condensed chromosomes. *Rbx1* knockdown with the appropriate siRNAs led to a decreased rate of first polar body extrusion and most oocytes were arrested at metaphase I. Moreover, downregulation of *Rbx1* caused accumulation of Emi1, an inhibitor of the anaphase-promoting complex/cyclosome (APC/C), which is required for mouse meiotic maturation. In addition, we found apparently increased expression of the homologue disjunction-associated protein securin and cyclin B1, which are substrates of APC/C E3 ligase and need to be degraded for meiotic progression. These results indicate the essential role of the SCF^βTrCP^-EMI1-APC/C axis in mouse oocyte meiotic maturation. In conclusion, we provide evidence for the indispensable role of RBX1 in mouse oocyte meiotic maturation.

## Introduction

Immature oocytes become fertilizable eggs through a process called meiotic maturation [1]. Various signal transduction pathways are involved in oocyte maturation, which is a multi-stage precisely orchestrated and orderly process [2,3]. Early oocytes are arrested in the diplotene stage of the ﬁrst meiotic prophase, until they are stimulated to undergo meiotic resumption at puberty. Meiotic resumption includes germinal vesicle breakdown (GVBD), chromosome condensation, spindle formation, and transition from meiosis I and meiosis II [4]. At the end of meiosis I, the first polar body (PB) is formed; the key to PB formation is the separation of homologous chromosomes [5].

Separation of homologous chromosomes is facilitated by a transient decline in M-phase promoting factor (MPF) activity at the time of transition from meiosis I to meiosis II [6]. MPF is a key kinase that catalyzes entry into the M-phase during meiosis I and meiosis II; it is composed of a catalytic subunit, p34cdc2, and a regulatory subunit, cyclin B1 [7]. Low MPF activity is necessary for germinal vesicle (GV) arrest; MPF is reactivated during GVBD [8]. MPF activity increases during the ﬁrst meiotic M-phase, where it is needed for spindle formation [7]. The synthesis and degradation of cyclin B1 is important for the control of MPF in mouse oocytes. In mouse oocytes, the synthesis of cyclin B1 increases progressively during meiotic maturation, reaching its maximum at the end of the ﬁrst meiotic M-phase. Cyclin B1 is degraded at the time of PB extrusion [9].

Cyclin B1 is degraded by the ubiquitin-proteasome pathway. The anaphase-promoting complex/cyclosome (APC/C), is a large mutlisubunit E3 ubiquitin ligase, catalyzes the formation of a ubiquitin chain on cyclin B1, which makes it a target for destruction by the 26S proteasome; this immediately results in inactivation of MPF [10]. Studies indicate that homologue disjunction in mouse oocytes is dependent on proteolysis of the separase inhibitor securin and cyclin B1, which are degraded by APC/C [11]. The Skp1-Cullin-F-box protein (SCF) complex is another very important E3 ubiquitin ligase [12]. By timely targeting of various substrates for degradation, the SCF complex regulates diverse cellular processes, including cell cycle progression, signal transduction, gene transcription, DNA replication, viral modulation, development, as well as circadian clock and protein quality control [13,14]. A basic component of SCF is RING box protein-1 (RBX1), also known as ROC1 (regulator of cullins-1). Cullin-1 is a scaffold protein, the N terminus of which binds to the Skp1-F-box complex, and the C terminus of which binds to RBX1 [15]. The core of the SCF complex comprises RBX1-cullins [16], whereas the substrate specificity of the SCF complex is determined by F-box proteins. RBX1 mediates the neddylation of Cul1, which activates SCF E3 ligase activity [17]. In a recent study, Skp1-Cul1–F-box/βTrCP (SCF^βTrCP^) was found to be responsible for Emi1(Early mitotic inhibitor 1) degradation in mouse oocytes. Emi1 inhibits the activity of APC/C, it undergoes SCF^βTrCP^-mediated destruction immediately after GVBD, which is necessary for progression through meiosis I [18]. RBX1, as one of the core components of the SCF complex, has been shown to play important roles in a range of cellular processes under physiological and pathological conditions, such as embryonic development, cell proliferation and cancer cell survival [19]; however, its role in the maturation of oocytes has not been confirmed.

In this study, we found that subcellular localization of RBX1 protein during mouse oocyte maturation changed dynamically and was closely associated with chromosome formation: we found that depletion of RBX1 decreased the rate of first PB extrusion (1^st^-PBE) and was accompanied by accumulation of Emi1

## Materials and Methods

All chemicals and culture media were purchased from Sigma Chemical Company (St. Louis, MO), unless stated otherwise.

### Ethics Statement

All experiments requiring the use of animals were approved by the Committee on the Ethics of Animal Experiments of Nanjng Medical University (Permit Number: 20080325).

### Antibodies

Rabbit polyclonal anti-RBX1 antibody (NB100-1672) was obtained from Novus Biologicals (Littleton, CO); mouse monoclonal fluorescein isothiocyanate (FITC)-labeled anti-α-tubulin antibody (ab13533), rabbit polyclonal anti-Emi1 antibody (ab18341), rabbit polyclonal anti-cyclin B1 (ab72), mouse monoclonal anti-securin (ab3305) and rabbit polyclonal IgG (ab27478) were purchased from Abcam (Cambridgeshire, UK).

### Oocyte collection and culture

Imprinting control region (ICR) white mice were maintained under a controlled environment with a temperature of 20–22°C, a 12/12-h light/dark cycle, 50–70% humidity, and ad libitum food and water. Oocytes arrested at the prophase stage, also called immature oocytes at the GV-stage, were collected from ovaries of 6- to 8-wk-old ICR female mice at 48 h after PMSG injection. Then, the oocytes were cultured in HCZB medium (Sigma, St. Louis, MO) at 37°C in an atmosphere containing 5% CO_2_. Only immature oocytes displaying a GV were cultured further in CZB medium (Sigma, St. Louis, MO) under liquid paraffin oil at 37°C in an atmosphere containing 5% CO_2_. At different times after culture, oocytes were collected for immunostaining, microinjection, or Western blot analysis.

### Nocodazole treatment of oocytes

Metaphase I and Metaphase II oocytes were treated with nocodazole, a microtubule-depolymerizing agent. For nocodazole treatment, 10 mg/ml of nocodazole in dimethyl sulfoxide (DMSO) stock was diluted in CZB medium to achieve a ﬁnal concentration of 20 µg/ml, and oocytes at the M-I (8 h of culture) and M-II (16 h of culture) stages were incubated for 10 min. After treatment, oocytes were washed thoroughly and ﬁxed for immunoﬂuorescence staining or were cultured for spindle recovery. In the control, oocytes were treated in the medium with the same concentration of DMSO but without nocodazole before examination. For spindle recovery, MII oocytes stages were cultured in CZB medium for 30 min after nocodazole treatment and then ﬁxed for immunoﬂuorescence staining.

### Immunohistochemistry

IHC staining for RBX1 was performed on deparaffinized and rehydrated Paraplast sections. The sections were immersed in 0.1% trypsin in CaCl_2_ solution for 30 min at 37°C to remove proteins and then incubated in 2 N HCl for 30 min. The slices were immediately soaked in 0.1% borax solution to stop the reactions and washed with tap water. Sections were immersed twice for 5 min in 10 mM citrate buffer (pH 6.0) and irradiated three times for 5 min in a standard microwave oven (750 W) to optimize IHC staining. Between the cycles, evaporated buffer was supplemented with hot distilled water. The slices were allowed to cool for 15 min at room temperature (RT) after heating in the microwave. Then, the sections were washed with Tris-buffered saline (TBS: 0.05 M Tris-HCl and 0.15 M NaCl [pH 7.6]) and the immunostaining procedure was started. Nonspecific staining was blocked twice, first with 3% H_2_O_2_ in methanol for 15 min to inhibit endogenous peroxidase activity, and then with 5% normal goat serum for 10 min at RT to block nonspecific sites. Next, the sections were processed for visualization of RBX1 using the IHC technique. All sections were incubated overnight at 4°C in a humidified chamber in the presence of anti-RBX1 antibody (1:100 dilution). Next, the slices were incubated for 20 min with biotinylated goat anti-rabbit IgG (1:200). Finally, sections were washed again in TBS and incubated for 10 min in a solution of streptavidin-ABC-HRP at a dilution of 1:100. Staining was performed in TBS containing DAB. Rabbit IgG was used for the negative control. Then, the sections were rinsed in tap water and counterstained with Mayer’s hematoxylin. The sections were examined under a microscope (Zeiss, Jena, Germany).

### Immunofluorescence detection and confocal microscopy

For single staining of RBX1 and α-tubulin, oocytes were fixed in 4% paraformaldehyde in PBS (pH 7.4) for at least 30 min at RT. After being permeabilized with 0.5% Triton X-100 at RT for 20 min, oocytes were blocked in 1% BSA-supplemented PBS for 1 h and incubated overnight at 4°C with 1:100 rabbit anti-RBX1 antibody or 1:200 anti-α-tubulin-FITC antibody, rabbit IgG was used in the place of anti-RBX1 antibody as the negative control. After washing three times with PBS containing 0.1% Tween 20 and 0.01% Triton X-100 for 5 min each, the oocytes were labeled with 1:100 FITC-conjugated goat-anti-rabbit IgG for 1 h at RT (for staining of α-tubulin, this step was omitted). After washing three times with PBS containing 0.1% Tween 20 and 0.01% Triton X-100, the oocytes were co-stained with propidium iodide (PI; 10 µg/ml in PBS). Finally, the oocytes were mounted on glass slides and examined under a confocal laser scanning microscope (Zeiss LSM 510 META; Zeiss, Germany). For double staining of RBX1 and α-tubulin, after RBX1 staining (the secondary antibody was 1:100 TRITC-conjugated goat-anti-rabbit IgG), the oocytes were again blocked in 1% BSA-supplemented PBS for 1 h at RT; this was followed by staining with 1:100 anti-α-tubulin-FITC antibody. Then, the oocytes were stained with Hoechst 33258 (10 µg/ml in PBS) for 20 min, mounted on glass slides, and examined under the confocal microscope.

### siRNA microinjection of GV oocytes

Two stealth siRNA duplexes of oligoribonucleotides targeting *Rbx1* were obtained from Invitrogen. The sequences were as follows: siRNA #1, 5′-UCCAUAAUGUGGUUCCUGCAGAUGG-3′; siRNA #2, 5′-AUACAAAGAUCCAUAAUGUGGUUCC-3′. Scrambled siRNA nucleotides were used as the negative control. The siRNAs were microinjected into GV-stage oocytes as described previously [20]. The final concentration of the control or *Rbx1*-specific siRNA was 20 µM, while non-injected GV oocytes served as the normal control group. In order to inhibit GVBD and ensure that the siRNAs had enough time to take effect, the microinjected oocytes were first cultured in CZB medium containing 50 µM IBMX for 12 h, and the knockdown efficiency of the two siRNA duplexes was assessed by real-time PCR analysis. For immunoblotting experiments, the oocytes were injected with the mixture of siRNA 1# and siRNA 2#, washed and transferred to IBMX-free CZB medium, and collected at different times during subsequent culture.

### Immunoblotting analysis

For each immunoblotting analysis, 700 mouse oocytes at the different stages of meiotic maturation (GVBD+2h, GVBD+6h, GVBD+10h) were separately collected in SDS sample buffer and heated for 5 min at 100°C. The proteins were separated by SDS-PAGE and then electrically transferred to polyvinylidene fluoride membranes. Following the transfer, the membrane were blocked in TBST (TBS containing 0.1% Tween 20) containing 5% skimmed milk for 2 h; this was followed by incubation overnight at 4°C with 1:500 rabbit polyclonal anti-RBX1 antibody, 1:200 rabbit polyclonal anti-Emi1 antibody, 1:500 rabbit polyclonal anti-cyclin B1 antibody, and 1:100 mouse anti-securin antibody or 1:2000 anti-β-tubulin (Abcam, Cambridge, MA) antibody. After washing three times in TBST for 10 min each, the membranes were incubated for 1 h at 37°C with 1:1000 horseradish peroxidase-conjugated goat anti-rabbit IgG or horseradish peroxidase-conjugated goat anti-mouse IgG. Finally, the membranes were processed using the enhanced chemiluminescence detection system (Amersham, Piscataway, NJ, USA).

### Statistical analysis

The differences between treatments were analyzed by paired Student’s *t*-tests using the SPSS software (SPSS Inc., Chicago, IL, USA). Before signiﬁcance analysis, all percentage data were subjected to arc-sine transformation. Data are expressed as mean ± SEM, and *P* < 0.05 is considered to indicate significance.

## Results

### Expression of RBX1 in mouse oocytes

**Figure 1:**
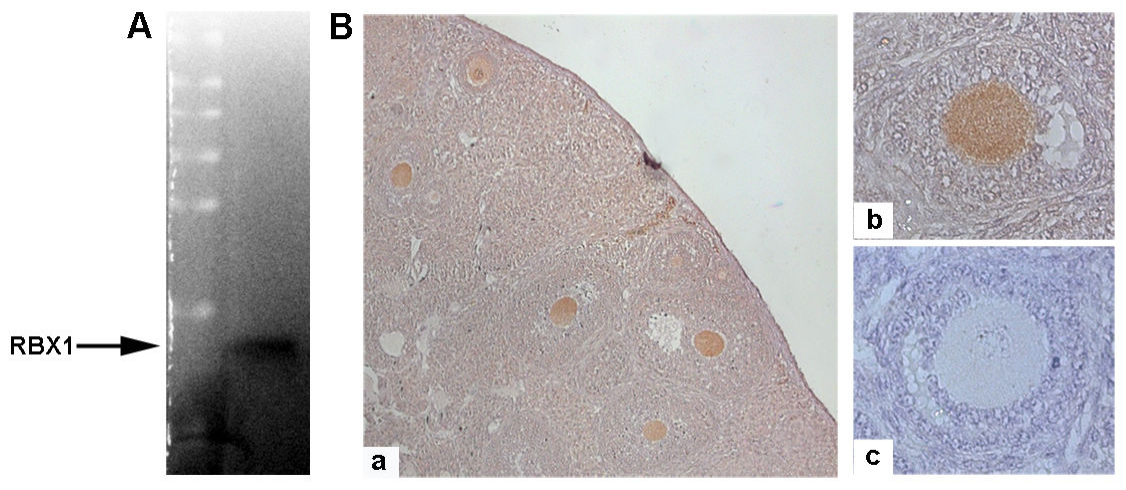
Presence of RBX1 in mouse oocytes. A) Western blot image showing the only band with the predicted molecular weight of 14 kD, indicating the presence of RBX1 in mouse oocytes. B) Immunohistochemistry images showing the abundant presence of RBX1 in oocytes (a. 10×, b. 40×, c. negative control).

Western blotting analysis using an anti-RBX1 antibody revealed an exclusive band at the expected molecular mass of 14 kDa (Figure 1A). Immunohistochemical staining showed the widespread distribution of RBX1 in mouse ovaries, with the highest signal detected in oocytes (Figure 1B,C). The negative controls did not show any signals in oocytes or granulosa cells (Figure 1D).

### Subcellular localization of RBX1 during oocyte meiotic maturation and its association with chromosomes and spindle

**Figure 2:**
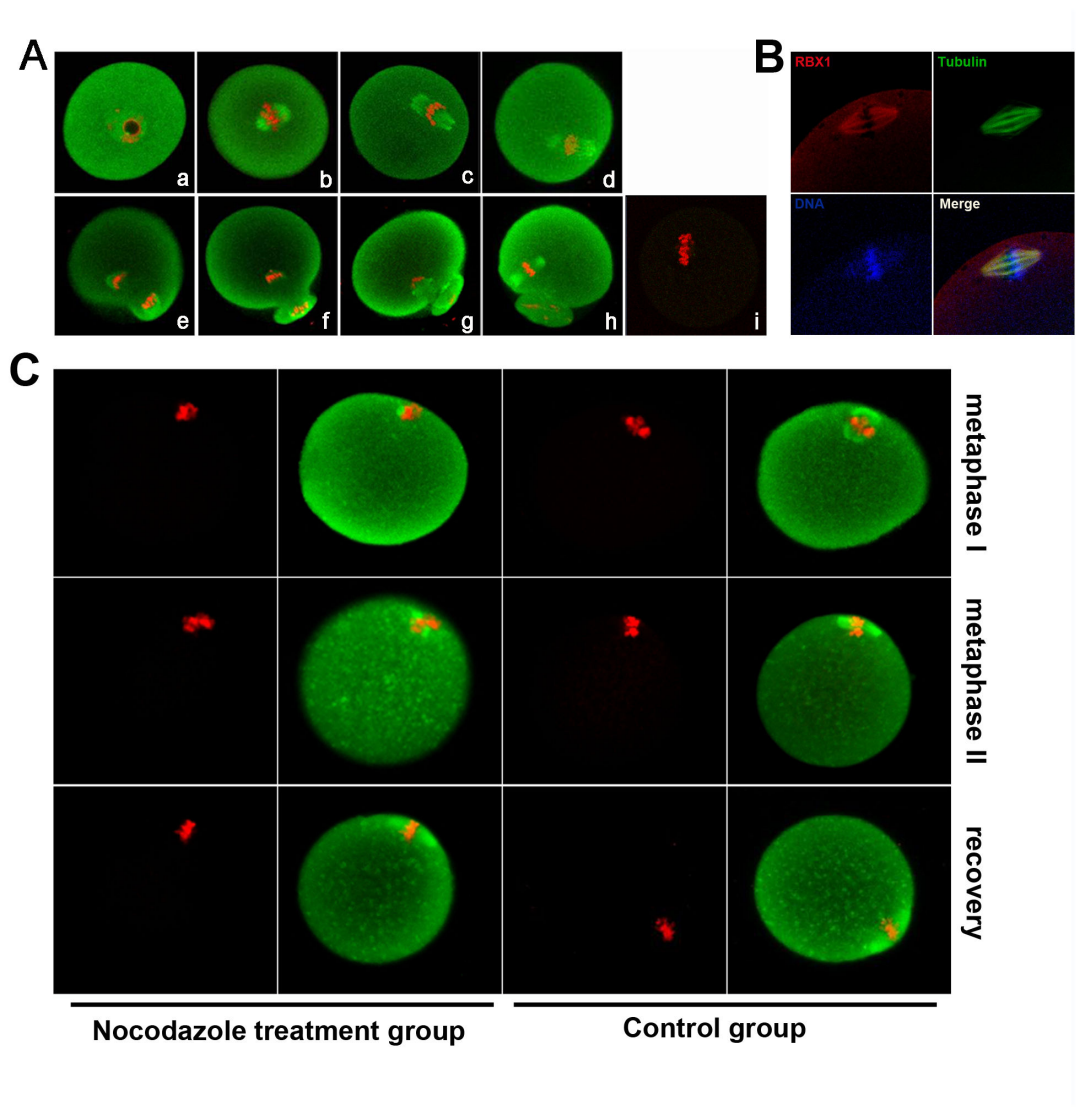
Subcellular localization of RBX1 during mouse oocyte meiotic maturation. A) Confocal microscope image showing immunostaining of RBX1 (green) and DNA (red) in oocytes at GV (a), GVBD+2 h (b), GVBD+4 h (c), GVBD+6 h (d), GVBD+8 h (e), GVBD+10 h(f), GVBD+12 h (g), and GVBD+16 h (meiosis II stage) (h), rabbit IgG was used as a negative control (i). B) Image of in vivo mature eggs double-stained with antibodies against RBX1 and α-tubulin: green, α-tubulin; red, RBX1; blue, chromatin; yellow, RBX1 and α-tubulin overlapping. C) Images of oocytes treated with nocodazole for spindle perturbation and then treated with fresh medium for spindle recovery. Oocytes at metaphase I and metaphase II stage were treated with nocodazole for spindle perturbation, and then MII oocytes were transferred to fresh CZB after washing away nocodazole for spindle recovery. Green, RBX1; red, DNA.

The dynamic distribution of RBX1 during mouse oocyte maturation is shown in Figure 2A. In GV-stage oocytes, RBX1 was localized in the entire oocyte and accumulated in the GV. With the development of oocytes, RBX1 was found to be concentrated around condensed chromosomes. At metaphase I, when the chromosomes were aligned at the equatorial plate, the RBX1 signal was found in the developing spindle. When the spindle started to migrate, uniform distribution of RBX1 was still found on the spindle; just before 1^st^-PBE, stronger accumulation of RBX1 was observed at the pole of the separating chromosomes. After extrusion of the first PB, RBX1 was again localized around the chromatin. Only faint background signal was visualized in MI oocyte immunostained by rabbit IgG as negative control, ensured the RBX1 signal on metaphase spindle was specific. This distribution was also confirmed by double staining with anti-RBX1 and α-tubulin antibody in meiosis II oocytes. As shown in Figure 2B, the RBX1 signals and α-tubulin signals were overlapping. The localization of RBX1 in mouse oocytes was also analyzed after treatment of the oocytes with a spindle-perturbing agent: metaphase I and metaphase II oocytes were treated with nocodazole, which resulted in disassembly of the microtubules. RBX1 disappeared from the chromosomes and diffused into the cytoplasm in the oocytes subjected to this treatment (Figure 2C). After spindle recovery, RBX1 accumulated in the chromosomes again (Figure 2C).

### Effect of Rbx1 knockdown on in vitro oocyte meiotic maturation

**Figure 3:**
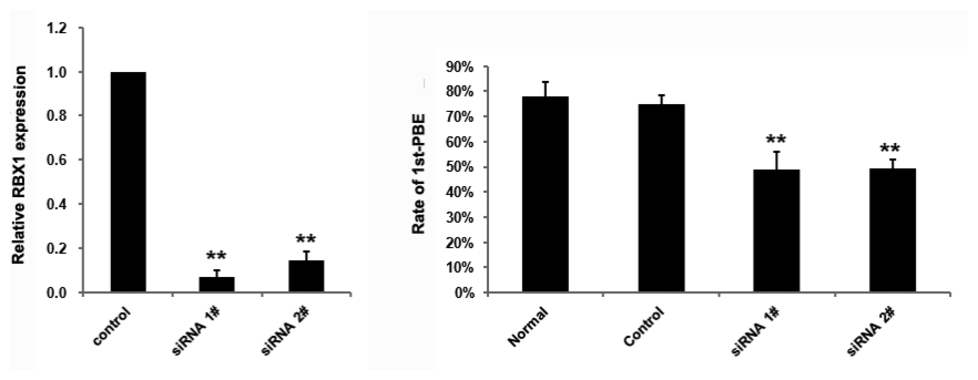
Knockdown of *Rbx1* in GV-stage oocytes. A) Real-time PCR results confirmed the downregulation of *Rbx1* expression. Error bars represent SD (*P < 0.01). The significantly declined ratio of 1st-PBE was also as a result of *Rbx1* knockdown, only oocytes with GVBD were calculated and counted, the results of five independent experiments are shown in Figure 3B (*P < 0.01).

To investigate the function of RBX1 in meiosis maturation, we knocked down *Rbx1* using specific siRNAs. Real-time PCR showed that the expression level of *Rbx1* was significantly reduced in the oocytes into which the siRNAs were microinjected (Figure 3A). In the *Rbx1* siRNA–injected group, GVBD was not affected (data not shown), however, the 1^st^-PBE rate was significantly lower than that in the control siRNA-injected group or normal culture group (Figure 3B). We further observed the oocytes that did not extrude the first polar body, analyzed the specific stages they arrested via immunostaining of α-tubulin and DNA, the results showed great majority of the oocytes were arrested in the progression of meiosis at metaphase I after *Rbx1* siRNAs microinjection (the precentage of oocytes arrested at GVBD, pro-MI and MI in siRNA 1# injected group (n=167) was 10.78%, 26.35% and 64.67%; in siRNA 2# injected group, the precentage (n=171) was 14.04%, 26.32% and 59.65% respectively).

### Accumulation of Emi1, securin and Cyclin B1 in Rbx1-knockdown oocytes

**Figure 4:**
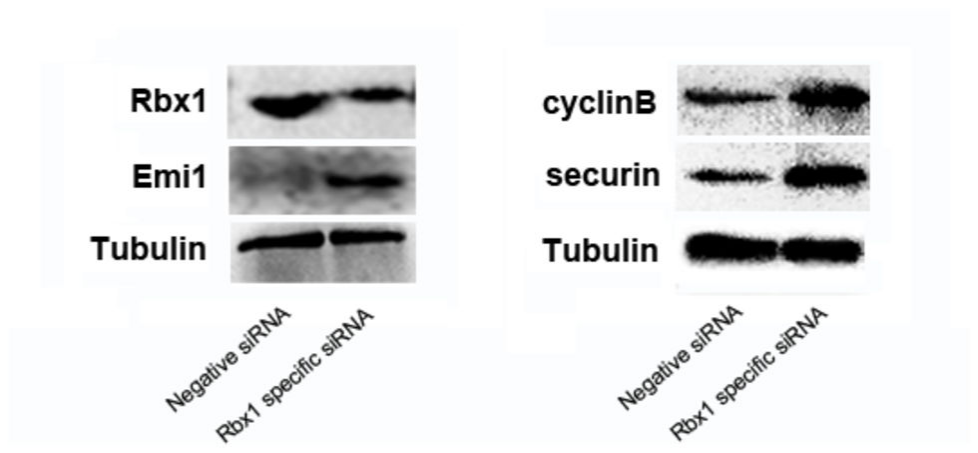
Western blot analysis for *Rbx1-*knockdown oocytes. The results confirmed the downregulation of RBX1 on protein level, and showed apparent increase in the expression of Emi1, cyclin B1 and securin.

*Rbx1*-specific siRNA-injected oocytes were collected at GVBD+2h to detect the protein level of RBX1, the result showed that RBX1 protein level was declined apparently, and subsequently the oocytes were collected at GVBD+6h to examine Emi1 protein expression; it showed visible accumulation of Emi1 in the *Rbx1*-knockdown group (Figure 4A). *Rbx1*-specific siRNA-injected oocytes were then collected at GVBD+10h to analyze the expression of securin and cyclin B1. The protein level of securin and cyclin B1 increased obviously in the *Rbx1*-downregulated oocytes (Figure 4B).

## Discussion

In this study, we have shown the expression and localization of RBX1 during mouse oocyte meiotic maturation. The localization of RBX1 in mammalian meiotic oocytes has not been reported previously, which makes this the first such report.

RBX1 displayed dynamic localization during mouse oocyte meiotic maturation, and its distribution was associated with the developing spindle and condensed chromosome. Stronger accumulation of RBX1 was observed at the pole of the separating chromosomes. After extrusion of the first PB, RBX1 was again localized around the chromatin. The characteristics of subcellular localization indicate that RBX1 has important functions in the process of mouse oocyte meiotic maturation. RBX1 distribution was closely associated with the spindle: when nocodazole was used to perturb the spindle assembly, RBX1 diffused into the cytoplasm, and on spindle recovery RBX1 was found around the spindle again. Downregulation of *Rbx1* by siRNA microinjection showed that the 1^st^-PBE rate declined in comparison with the control groups and most oocytes were arrested at metaphase I, which indicates disruption of transition from meiosis I to meiosis II, provided direct evidence for the involvement of RBX1 in mouse oocyte meiotic maturation.

Since RBX1 is a key component of the SCF E3 ubiquitin ligases, required for SCF to direct a timely degradation of diverse substrates, it was speculated that the important role of RBX1 during mouse oocyte meiotic maturation was brought about by destruction of specific SCF substrate. Emi1, an inhibitor of APC/C was noticed as a specific substrate of SCF^βTrCP^, which has essential functions in mouse oocyte maturation [21]. Emi1 is reportedly present at the GV-stage and destroyed rapidly following GVBD, Emi1 destruction is necessary for subsequent meiotic maturation, microinjection of exogenous Emi1 shows that excess Emi1 causes inhibition of the MI→MII transition [18]. This is consistent with the effect of *Rbx1* knockdown, and the Emi1 accumulation following *Rbx1* knockdown demonstrated the hypothesis that RBX1 played role in oocyte meiotic progression via regulating Emi1 degradation. APC/C is a highly conserved E3 ligase complex that mediates the destruction of key regulatory proteins during multiple cell cycle transitions, including the exit from mitosis and metaphase-anaphase transition [22,23]. In the progression of mouse oocyte maturation, disjunction of homologous chromosomes in meiosis depends on the APC/C activity [24]. The activation of APC/C requires the binding of a cofactor, either Cdh1 or Cdc20. APC/C^cdh1^ was active first, during early prometaphase I, and then was replaced by APC/C^cdc20^ during late meiosis I, APC/C^cdc20^ in oocytes proteolytically degrades securin and cyclin B1, drives the anaphase I onset and PBE [25–27]. The degradation of separase inhibitor securin and the MPF regulatory subunit cyclin B1 in mouse oocytes begins ~2-3h prior to extrusion of the first PB, is necessary required for homologue disjunction [11,28]. Polar body formation was inhibited by microinjection of exogenous securin and cyclin B1 mRNA, DNA staining demonstrated that homologous maitained a metaphase or metaphase-like configuration [11], it coincided with our observation that great majority of oocytes arrested at metaphase I after *Rbx1* knockdown, excess level of cyclin B1 and securin we found before PBE alluded that they were downstream effector of RBX1, through regulating their destruction, RBX1 performed critical function in MI→MII transition. In summary, our study demonstrated the essential role of RBX1 in the meiotic progression of mouse oocyte. The RBX1 protein in mouse oocyte, as a RING subunit of SCF E3 ubiquitin ligases, was required for SCF^βTrCP^ to direct a timely degradation of Emi1 between GVBD and MI. *Rbx1* knockdown resulted in Emi1 accumulation, the elevated level of Emi1 led to inhibition of APC/C^cdc20^ activity. Low APC/C^cdc20^ activity prevented the degradation of securin and cyclin B1, which is indispensable for homologous chromosome separation and anaphase onset. The increased securin and cyclin B1 before PBE finally resulted in defective transition from meiosis I to meiosis II. Both classic E3 ubiquitin ligases – SCF and APC complex – are closely associated with regulation of oocyte meiotic maturation, and Emi1 is a link protein between the two. The SCF^βTrCP^-EMI1-APC/C axis has been proven to play critical role of in genome reduplication [29,30]. Our findings provided evidence that the SCF^βTrCP^-EMI1-APC/C axis is present in the mouse oocyte and plays a crucial role in regulating mouse oocyte meiotic maturation.

RBX1 is evolutionarily conserved from plants to mammals, with 86% homogeneity between mice and humans [31,32]. Thus, we presume that RBX1 may play a similar role in human beings. Moreover, the role of the SCF^βTrCP^-EMI1-APC/C axis should be further investigated, especially in clinical in vitro oocyte maturation techniques, which are yet to be established as a mainstream fertility treatment because of the lower chances of live birth compared with conventional in vitro fertilization techniques.
